# Neutrophils and aortic medial amyloid: mutually beneficial or a dangerous combination?

**DOI:** 10.3389/fimmu.2026.1699039

**Published:** 2026-02-06

**Authors:** Alana Maerivoet, Sarah Shirley, Rebecca Price, David Wilkinson, Helen L. Wright, Jillian Madine

**Affiliations:** 1 Institute of Systems, Molecular and Integrative Biology, University of Liverpool, Liverpool, United Kingdom; 2 Department of Cardiothoracic Surgery, Liverpool Heart and Chest Hospital, Liverpool, United Kingdom; 3 Institute of Life Course and Medical Sciences, University of Liverpool, Liverpool, United Kingdom

**Keywords:** aortic medial amyloid, aortic pathologies, inflammation, neutrophil, protein aggregation

## Abstract

**Introduction:**

Amyloid deposition and inflammation are associated with many human diseases, with inflammatory cells found co-localised with amyloid in a range of tissues. Medin is the peptide that forms the most common localised amyloid in the aortic medial layer, yet remarkably little is known about its role in disease or factors that modulate its aggregation.

**Methods:**

We investigated the effect of neutrophil degranulation supernatants on medin fibril formation *in vitro* and explored the impact of inhibiting proteolytic components of neutrophil degranulation on aggregation using Thioflavin T fluorescence, transmission electron microscopy and cell viability analyses.

**Results:**

We showed that neutrophil supernatants reduced fibril formation of medin and its associated cell toxicity. Addition of inhibitors targeting MMPs (GM6001) and serine proteases (AEBSF) reversed the effects of neutrophils on fibril formation and cell toxicity. In contrast, inhibiting cysteine proteases using E64 showed comparable low ThT fluorescence and a lack of fibrils similar to what is observed for medin in the presence of neutrophil supernatants alone. However, despite appearing comparable to neutrophils alone, species produced showed significantly increased cell toxicity of up to 60% (P<0.0001).

**Discussion:**

This data has implications for understanding the role of neutrophil-mediated inflammation in medin-associated pathologies and provides avenues to explore for future therapeutic intervention.

## Introduction

Aortic medial amyloid (AMA) is the most common form of localised amyloid found in the medial layer of the thoracic aorta in up to 100% of the Caucasian population over the age of 50 (1, 2). Several studies have indicated that AMA may play a pathogenic role in thoracic aortic pathologies such as aneurysm and dissection (3, 4) and cerebrovascular diseases including vascular dementia, cerebral amyloid angiopathy and Alzheimer’s disease (AD) (5, 6). Medin is a 50 amino acid peptide cleaved from milk fat globule EGF-like factor 8 protein (MFGE8) that forms the main protein component of AMA (7) but with no clear mechanism for cleavage currently elucidated.

Neutrophils are a key part of the acute inflammatory response and the first line of cellular defence against pathogens such as bacteria and fungi. They have several functions including production and release of cytokines/chemokines, proteases and other inflammatory factors, phagocytosis and production of neutrophil extracellular traps (NETs) and reactive oxygen species (ROS) (8). Neutrophils and neutrophil-derived proteases, ROS and NETs are increasingly being identified as important mediators of cardiovascular diseases including aortic pathologies (9, 10). Elevated levels of neutrophils and neutrophil to lymphocyte ratio (NLR) are associated with poorer clinical outcomes in many diseases including aortic aneurysm and dissection, and have been proposed as potential prognostic biomarkers (11, 12). Additionally, cognitive decline in AD has been associated with higher NLR levels (13).

Many amyloid diseases including AD are associated with inflammation (14). The presence of amyloid causes local tissue damage and induces an inflammatory response. In turn, an inflammatory environment can initiate the process of amyloid formation generating a self-perpetuating cycle and chicken-egg dilemma for the initiating factor. We have previously shown that medin can induce pro-inflammatory activation of vascular smooth muscle cells and endothelial cells (15–18). Given the association of AMA with aortic pathologies (3, 4) and the strong evidence of poorer outcomes in these conditions upon increased white blood cells, neutrophils and NLR levels (19), here we set out to explore the relationship between neutrophil response and AMA further. We evaluated the effect of neutrophil presence and activation state on medin fibril formation *in vitro*. Additionally, we examined mechanisms underlying this effect by investigating the role of proteases released by degranulating neutrophils and probed the effect of protease inhibition relative to medin toxicity as a potential pathological mechanism in vascular diseases.

## Methods

### Neutrophil isolation and degranulation

Blood was collected from 2 volunteers under written informed consent in accordance with the declaration of Helsinki and following the recommendations of the University of Liverpool Central University Research Ethics Committee D for healthy controls (Ref. 10956). Neutrophil isolation was performed according to the method previously described (20). Following this method, whole blood drawn into lithium-heparin vacutainers was decanted into a 50 mL falcon tube. HetaSep reagent was added to the blood in a ratio of 1:5 (HetaSep: blood). This was mixed by inversion and incubated at 37°C for 30 minutes. Following sedimentation, the buffy coat was removed, layered 1:1 on top of Ficoll-Paque and centrifuged at 500 g for 30 minutes. The supernatant, containing the peripheral blood mononuclear cells, plasma and residual Ficoll-Paque, was removed and the neutrophil pellet was resuspended in a small volume of media and transferred to a clean falcon tube. The resuspended neutrophils were washed in 40 mL media and centrifuged at 500 g for 3 minutes. Again, the supernatant was removed, and the neutrophil pellet was resuspended in 3 mL media. Next, ammonium chloride lysis buffer was added 1:9. Following inversion 2–3 times and 3 minutes incubation at room temperature, the neutrophils were centrifuged at low speed (1300 rpm) for 5 minutes, the supernatant discarded, and the cells resuspended in 3 mL media for counting. Cells were counted in a BioRad TC20 cell counter, and the cell count was adjusted to 5x10^6^/mL. Neutrophil purity was assessed by cytospin and was routinely >97%. Representative neutrophil image in **Supplementary Figure S1**.

The isolated neutrophils were treated to encourage neutrophil degranulation by adding granulocyte macrophage colony stimulating factor (GM-CSF) (5 ng/mL) at 37°C for 30 minutes in a rotating incubator. Following incubation, cytochalasin B (6 μg/mL) and N-formylmethionine-leucyl-phenyalanine (fMLP) (1 μM) were added and the neutrophils were incubated at 37°C for a further 30 minutes. Control (untreated) neutrophils were incubated in media at 37°C for 60 minutes. Following treatment, the cells were centrifuged at 1000 g for 3 minutes and the supernatant collected and stored at -20°C until needed.

### Recombinant medin expression

A medin construct with an N-terminal His-SUMO tag in a pOPINS vector (OPPF) was transformed into Lemo 21 (DE3) cells (New England Bioscience, Hitchin, UK) and grown at 37°C. Cells were induced at OD_600_ 0.8–1.0 with 0.5 mM IPTG for 16 h at 18°C. After harvesting, cells were resuspended in 6 M guanidine hydrochloride (GdmCl), 20 mM sodium phosphate, 500 mM NaCl, 20 mM Imidazole pH 7.4, and frozen at −20°C. Cells were homogenised and the clarified lysate loaded onto a 5 mL Ni^2+^-NTA column and washed with 10 column volumes (CV) 6 M GdmCl. Medin fusion protein was eluted with 3 CV of 6 M GdmCl, 20 mM sodium phosphate, 500 mM NaCl, 250 mM Imidazole pH 7.4, and stored at −20°C. The fusion protein was buffer exchanged into PBS, pH 7.4, and the His6-SUMO tag removed by incubation with SUMO protease I at 4°C for 1 h. The cleavage mixture was passed through a 5 mL Ni^2+^-NTA column and the flow-through containing medin collected. Purity was assessed to be >95% via SDS-PAGE.

### Thioflavin T fluorescence

Thioflavin T (ThT) fluorescence assays were carried out using a Flexstation 3 microplate reader (Molecular Devices) in sealed 96-well, black-walled, clear-bottomed microplates (Nunc). Data were recorded every 5 min using bottom read mode, with excitation at 440 nm and emission at 490 nm. The assay was carried out using 20 µM medin alone and co-incubated with 5, 10, 15 and 20% v/v of untreated or treated neutrophils in PBS with 2 µM ThT at 37°C under quiescent conditions with 5 s shaking every 5 mins for up to 24 hours. Experiments were carried out using neutrophils isolated from 2 donors with 3 wells per condition. A second experiment was conducted using 20 µM medin in the presence of 10% v/v of one donor in the presence and absence of protease inhibitors at final concentrations of 100 µM GM6001, 1.25 mM 4-(2-aminoethyl)-benzenesulfonyl fluoride (AEBSF) and 40 µM E64 (all Bio-Techne) with 3 wells per condition. Control neutrophil, inhibitor, DMSO (0.2% final volume as needed to dissolve GM6001) and PBS spectra were also included. ThT data was analysed using ANOVA with Fisher’s Least Significant Difference *post hoc* test.

### Transmission electron microscopy

Endpoint samples from ThT experiments were analysed by transmission electron microscopy (TEM). 5 μL of sample was mounted onto a carbon-coated copper grid for 2 min, blotted and stained with 2% (w/v) uranyl acetate for 30 s, washed with water three times before being allowed to dry. Images were collected on a 120 kV Tecnai G2 Spirit BioTWIN electron microscope (Thermo Fisher Scientific) with a SIS Megaview III camera.

### Protease screening

Proteases present in the NDP were assessed using R&D systems proteome profiler human proteases array kit (ARY021B) as per manufacturer’s instructions. Briefly, array membranes were blocked in 2 mL Array Buffer 6 for 60 min with rocking. Treated and untreated neutrophils (n=2) were prepared by diluting 250 μL in 1250 μL diluent to make a final volume of 1500 μL. Next, 15 μL of Protease Detection Antibody Cocktail was added to each prepared sample, mixed and incubated at room temperature for 60 min. Following blocking, Array Buffer 6 was aspirated from each well and the prepared sample/antibody mixtures were added to each membrane and incubated overnight at 4°C with rocking. Following incubation, membranes were washed in 20 mL of wash buffer for 10 min, for a total of three washes. After washing, membranes were incubated for 30 min at room temperature in 2 mL Streptavidin-HRP with rocking. The washing step was repeated for 10 min three times. Finally, the membrane was placed in a clear plastic window and 1 mL of the prepared Chemi Reagent Mix was carefully and evenly added to the membrane before covering and imaging using a BioRad Chemidoc Imaging System. Images were taken at 10 s, followed by 10 more images over 10 min. The best image was selected and subjected to pixel analysis using BioRad Image Lab volume tools. Raw pixel counts were analysed visually in R studio pheatmap to generate a heatmap. Proteases with pixel counts greater than 90,000 were visualised in ggplot2.

### MMP activity assay

Protease activity was assessed by fluorescence assay. 10 μL treated and untreated neutrophils (n=2) were diluted with 70 μL F56 Buffer (100 mM Tris pH 7.5, 150 mM NaCl, 0.05% Brij35, 0.1% PEG 6000, 10 mM CaCl_2_). Next, the mixture was warmed to 37°C, before 20 μL of the fluorescent FS-6 MMP substrate was added (final concentration 10 μM) and fluorescence measured at λ_ex_360 nm and λ_em_440 nm on a FluoStar Optima plate reader (BMG Labtech). Data were recorded at 30 s intervals for 30 min, and linear reaction rates taken to measure reaction velocity.

### Cell toxicity

Human umbilical vein endothelial (HUVEC) cells were maintained in endothelial cell growth medium (Promocell). Cells were added to 96-well plates at 6,000 cells per well in 80μL and incubated at 37°C with 5% CO_2_ for 24 h. Medin fibrils were formed at 20 µM alone and in the presence of 10% v/v untreated neutrophils from one donor with and without inhibitors at final concentrations of 100 µM GM6001, 1.25 mM AEBSF and 40 µM E64 in PBS to match ThT conditions, pH 7.4 with stirring at 37°C for 7 days. Control neutrophils, inhibitors and DMSO were also set up. 20 µL of sample (n=6 per group) were added to the cells and incubated for 24 h. After this time, 10 μL of Cell Counting Kit-8 (CCK-8, Stratech) was added to each well, and the absorbance recorded at 450/600nm over 3 h. 600nm reference wavelength was used to correct for the addition of insoluble fibrillar material (as per CCK-8 dye instructions). Data were processed and analysed in Origin Lab to report % viability in comparison with live (buffer alone) and dead (1% triton final concentration) controls using one-way ANOVA with Fisher’s Least Significant Difference *post hoc* test following removal of any obvious outliers to always retain n≥3.

## Results

### Neutrophils prevent medin fibril formation in vitro

**Figure 1:**
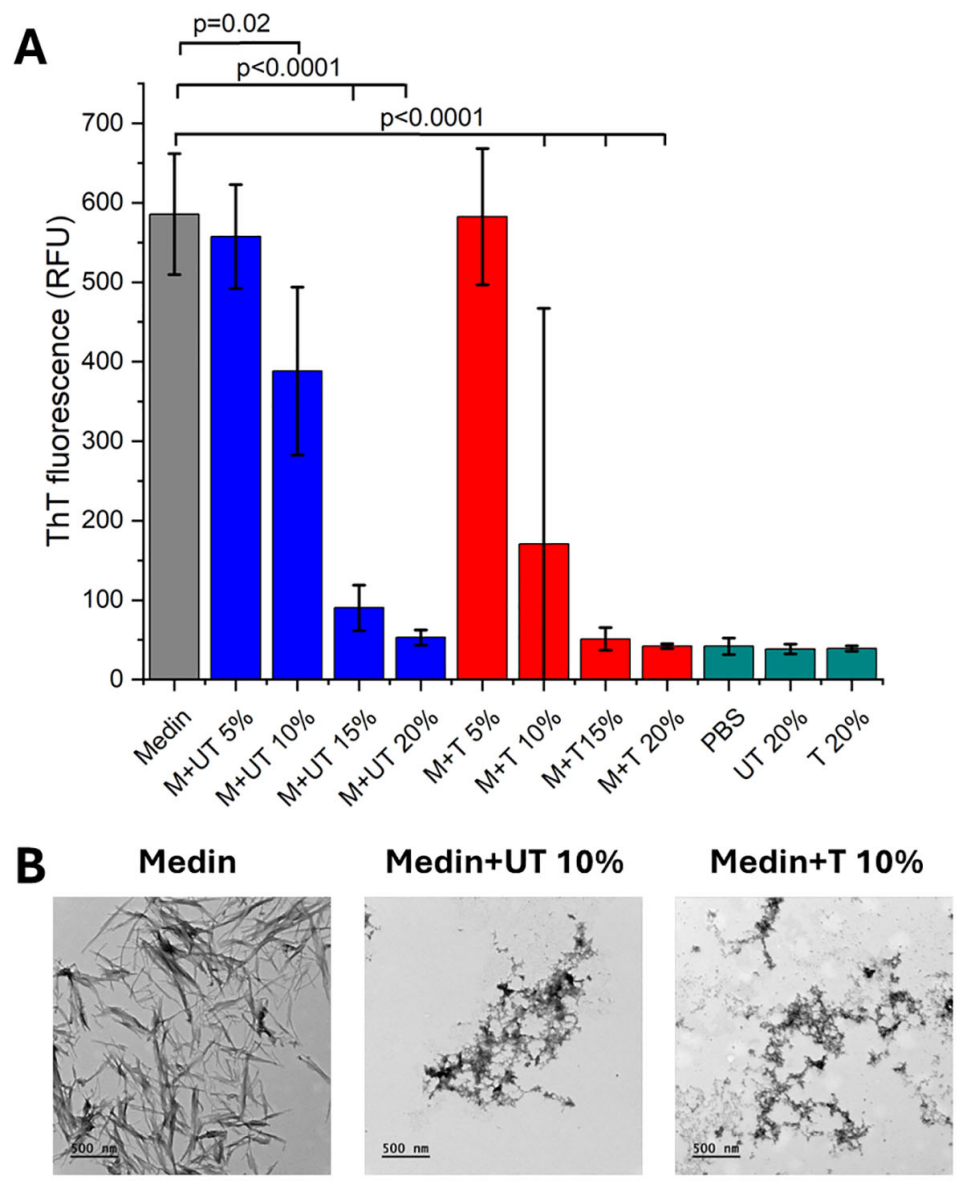
Neutrophil supernatants prevent fibril formation in a dose dependent manner. **(A)** Thioflavin T fluorescence after 24 hrs of incubation for 20 µM medin alone (M, grey) in PBS, pH7.4 and in the presence of untreated (UT, blue) and treated (T, red) neutrophil supernatants at 5, 10, 15 and 20% v/v as indicated, with PBS and UT/T controls alone at 20% v/v also shown (dark cyan). Data is presented as mean ± SD for n=3 per condition with neutrophil supernatants from 2 different people with p-values determined by ANOVA with Fisher *post-hoc* analysis indicated. **(B)** Corresponding transmission electron micrographs following incubation under the conditions as shown. Scale bar is 500 nm.

Medin aggregates to form fibrillar species after 24 hours observed by an increase in ThT fluorescence (**Figure 1A**) and TEM analysis (**Figure 1B**). Addition of increasing amounts of neutrophil supernatants showed a concentration-dependent reduction in ThT fluorescence suggesting prevention of fibril formation by medin (**Figure 1A**). It was also noted that treated neutrophil supernatants had a lower ThT fluorescence value than untreated for 10-20% addition, with presence of 10% neutrophil supernatant showing a significant difference (p=0.002). Control neutrophil supernatants alone (in the absence of medin) did not show any change in ThT fluorescence compared with PBS alone. A reduction in fibril formation in the presence of 10% neutrophil supernatant was confirmed by TEM analysis with largely pre-fibrillar species observed in the presence of neutrophil supernatants (**Figure 1B**).

Neutrophil supernatants were also added to pre-formed fibrils and effect on fibril presence assessed via ThT fluorescence for up to 4 days. Treated neutrophil supernatants showed a change in ThT profile after 2 days post-addition suggesting re-arrangement of fibrils to form a species with altered ThT binding occurred (**Supplementary Figures S2A, B**). This was not observed in the presence of untreated supernatants. TEM confirmed altered fibril morphology/association in the presence of treated neutrophil supernatants but not untreated (**Supplementary Figure S2C**).

### Neutrophil supernatant composition and evaluation of inhibitors

**Figure 2:**
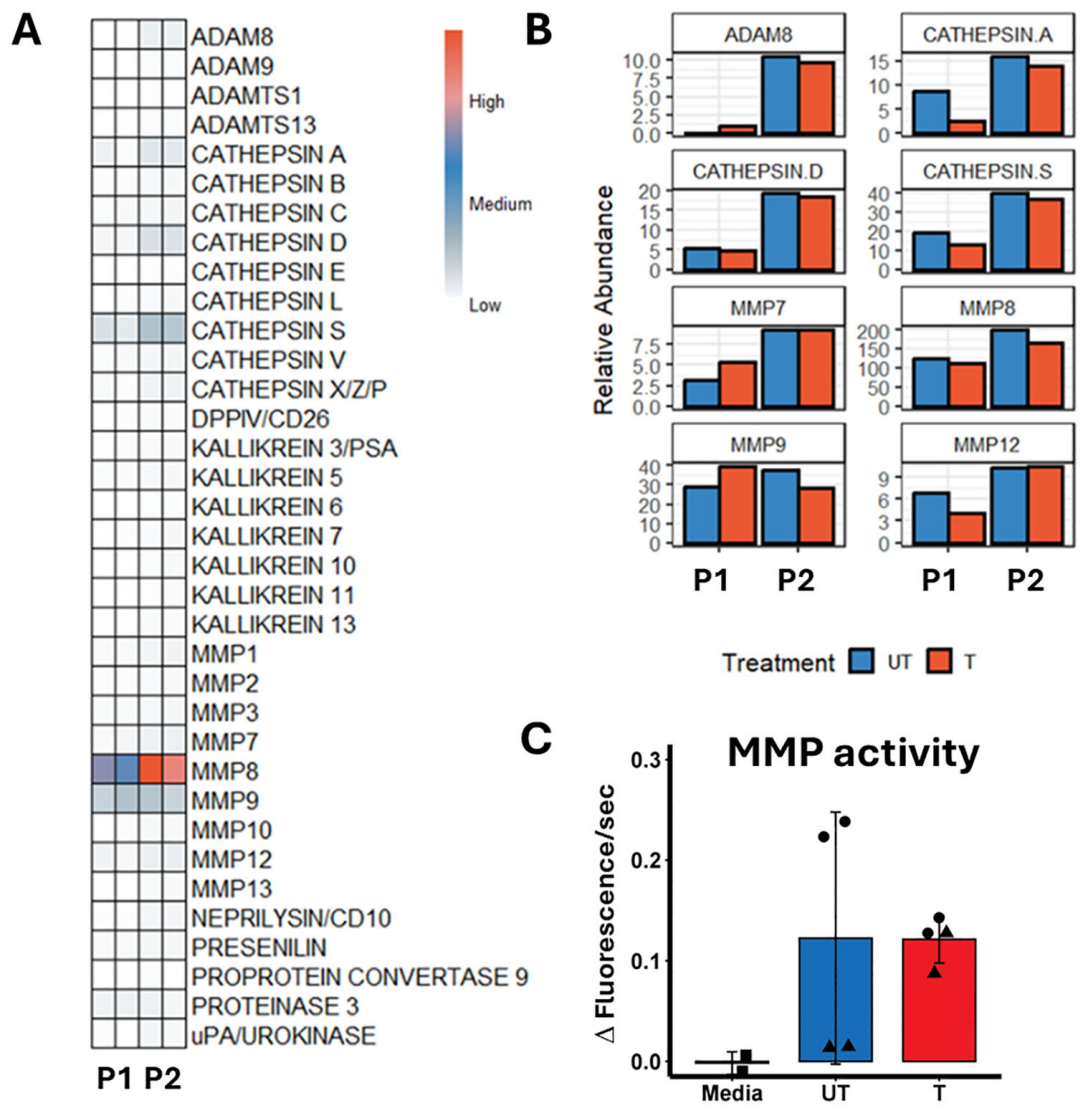
Analysis of neutrophil supernatants identified highly abundant proteases. **(A)** Protease array panel summary for neutrophil supernatants from the 2 donors used (P1 and 2) treated (T) and untreated (UT). **(B)** Relative abundance graphs for proteases where pixel counts were above 90,000. **(C)** MMP activity assay, fluorescence data shown as mean ± SD with duplicate data for 2 donors (P1-triangles, P2-circles).

We next evaluated the composition of the neutrophil supernatants using a protease array. This revealed that matrix metalloproteinases (MMPs) 7, 8, 9 and 12 together with cathepsins A, D and S and ADAM8 (A Disintegrin and metalloproteinase domain-containing protein 8) had the highest relative abundance (**Figures 2A, B**). Additionally, enzymatic assays showed MMP activity in the supernatants (**Figure 2C**). We used supernatants from neutrophils that were untreated (incubated for an hour following isolation) and treated (forced to degranulate). Our protease array data showed that even without actively stimulating degranulation the neutrophils still released proteases into the culture supernatant.

To assess the contribution of the identified proteases on the observed inhibition of fibril formation in the presence of neutrophil supernatants we selected GM6001 as a broad MMP inhibitor, AEBSF (4-(2-aminoethyl)benzenesulfonyl fluoride hydrochloride) as a serine protease inhibitor and E64 as a cysteine protease inhibitor to determine the contribution of different protease families.

**Figure 3:**
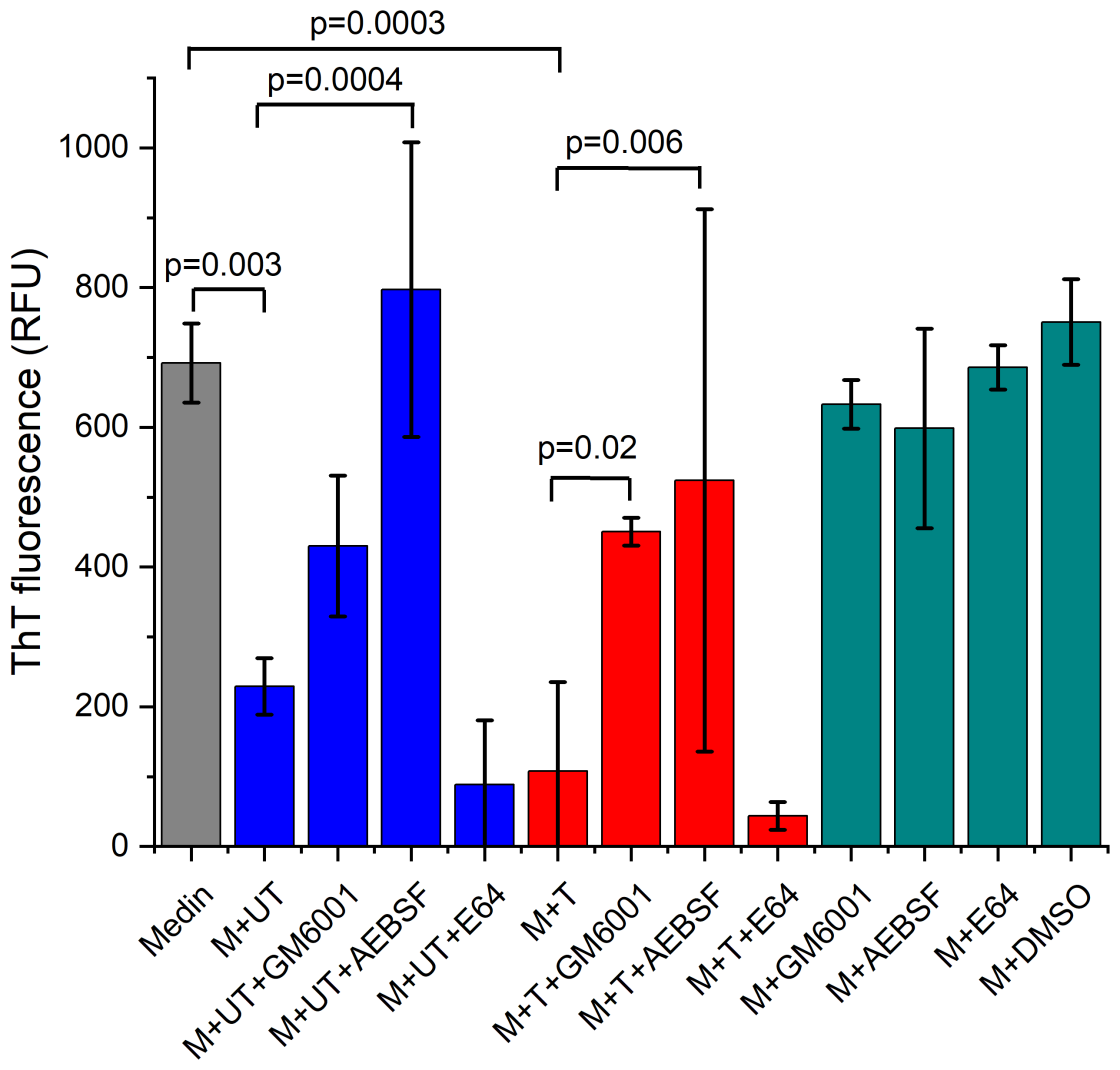
Thioflavin T fluorescence assay in presence of inhibitors GM6001 and AEBSF restores fibril formation. Thioflavin T fluorescence after 24 hrs of incubation for 20 µM medin alone (grey) in PBS, pH 7.4 and in the presence of untreated (UT, blue) and treated (T, red) neutrophil supernatants from P1 at 10% v/v, n=3 per condition and in the presence of inhibitors GM6001, AEBSF and E64 as shown. Control inhibitors and DMSO added to medin are also shown (dark, cyan). Data presented as mean ± SD with p-values determined by ANOVA with Fisher *post-hoc* analysis indicated.

Inhibitor concentrations were determined using previous studies reporting activity and cell viability data (21) together with aqueous solubility limits. Concentrations of 100 µM GM6001, 1.25 mM AEBSF and 40 µM E64 were used. ThT fluorescence using treated and untreated neutrophil supernatants from one person (P1) was repeated in the presence and absence of selected inhibitors. A significant difference in the ThT fluorescence was observed in the presence of untreated and treated neutrophil supernatants at 10% v/v consistent with data presented in **Figure 1** (**Figure 3**, p=0.003 and 0.0003 respectively) with treated having lower ThT fluorescence than untreated. Presence of GM6001 partially restored fibril formation for both treated and untreated neutrophil supernatants. The presence of AEBSF had a greater effect on restoration of fibril formation with some samples showing comparable ThT fluorescence to medin alone. In contrast, the presence of E64 showed low ThT fluorescence.

**Figure 4:**
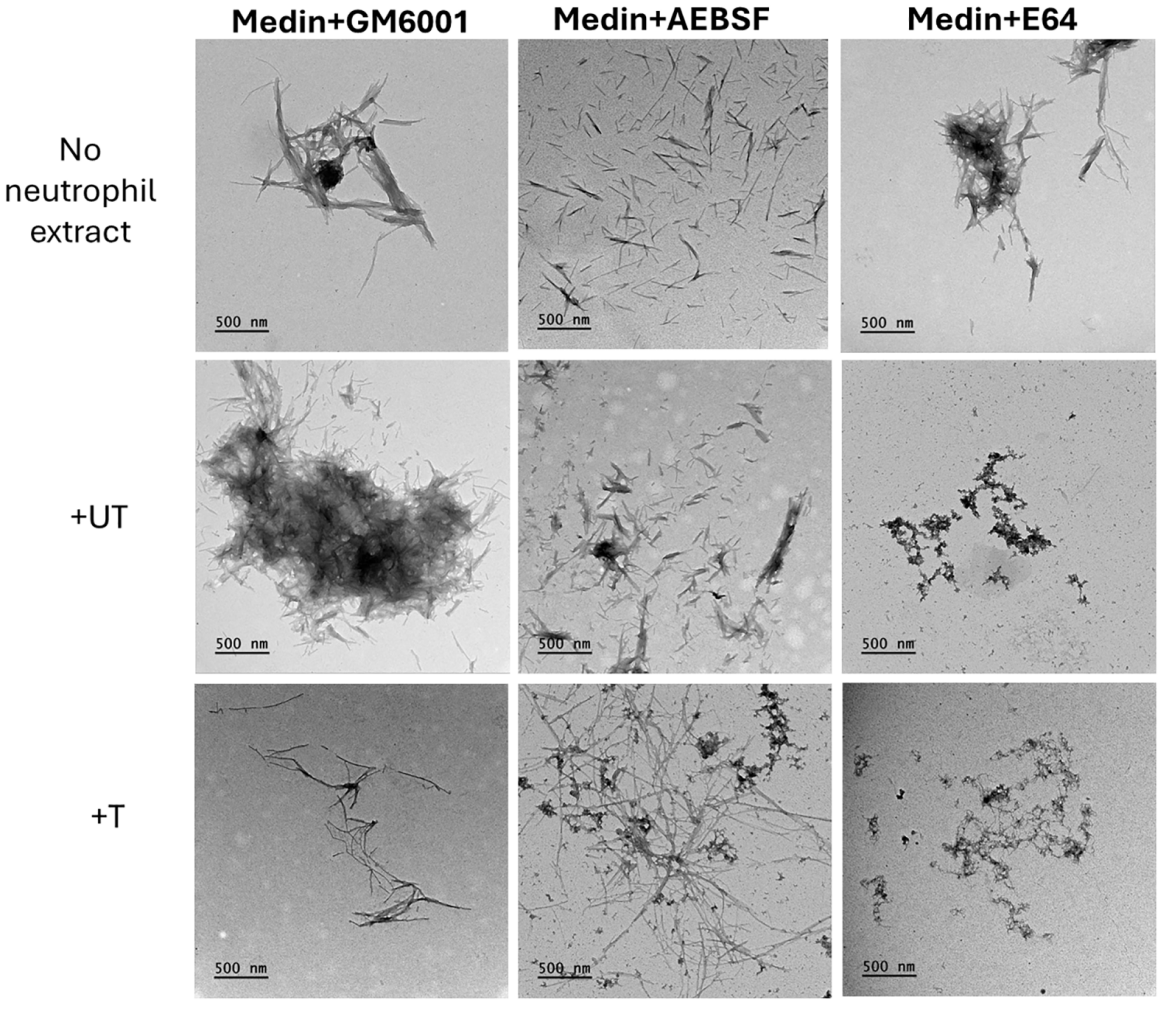
Transmission electron micrographs showed altered aggregate morphology for medin in the presence of inhibitors. Representative images for medin aggregates formed in the presence of neutrophil supernatants and inhibitors as indicated. Scale bar is 500nm.

TEM in the presence of neutrophil supernatant and inhibitors showed that addition of GM6001 and AEBSF can restore some of the fibril formation ability of medin (**Figure 4**). However, the morphology and distribution of the fibrils formed particularly in the presence of AEBSF are more spread out and distinct compared with medin alone. In contrast, in the presence of E64 and neutrophil supernatant no fibrils were observed with pre-fibrillar aggregates showing association in a network-like arrangement but lacking defined fibril morphology present instead. Fibrils were observed for all conditions when medin was incubated in the presence of inhibitors alone.

### Is this beneficial for pathogenicity?

**Figure 5:**
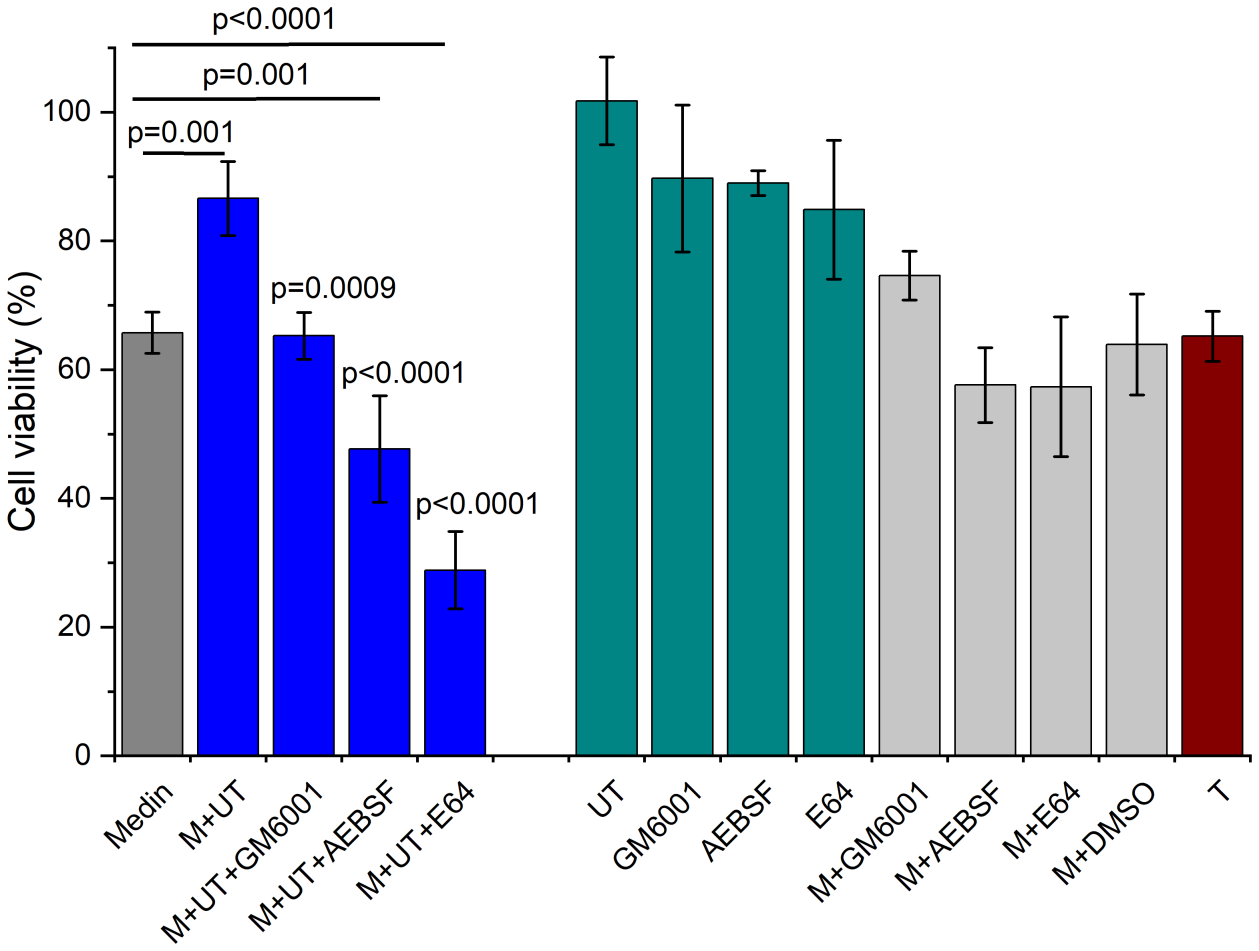
Cell viability of HUVECs showed altered viability in the presence of neutrophil supernatants and inhibitors. Data is shown following normalising to live and dead controls for medin alone (dark grey), in the presence of untreated (UT) neutrophil supernatants with and without inhibitors (blue), UT and inhibitors alone (dark cyan), medin plus inhibitors (light grey) and treated alone (T, dark red). Data is presented as mean ± SD with p-values determined by ANOVA with Fisher *post-hoc* analysis between medin alone indicated by lines, and between M+UT alone and M+UT plus inhibitors indicated by p values above columns, n=3–6 per condition.

Cell viability assessment showed ~66% cell viability upon exposure to medin fibrils formed alone (**Figure 5**). Treated neutrophil supernatants displayed reduced cell viability of ~35% alone possibly due to one of the degranulation reagents used or inherent neutrophil activity (**Figure 5**, dark red) so the remaining viability data presented is for untreated neutrophil supernatants only. Addition of fibrils pre-formed in the presence of 10% neutrophil supernatant showed a higher viability of ~87% compared with medin incubated alone (p=0.001). When medin was incubated in the presence of neutrophil supernatants in combination with inhibitors reduced cell viability was observed compared with medin and neutrophil supernatants alone (GM6001 p=0.0009, AEBSF and E64 p<0.0001). The presence of AEBSF and E64 also significantly lowered cell viability compared with medin alone (p=0.001 and p<0.0001 respectively). Medin and inhibitors alone (or DMSO control) did not show any significant difference to medin alone. Control experiments of inhibitors showed cell viabilities of 85-90% with untreated neutrophil supernatants alone having no observed effect on viability.

## Discussion

There are a range of factors that can influence fibril formation both *in vitro* and *in vivo* and could be responsible for initiation or modulation of disease-associated pathogenic fibril formation, including pH and co-factors that promote or inhibit aggregation. In turn, the presence of fibrils can induce a series of pathways and other processes that alter the local environment. Deciphering which comes first is a challenging phenomenon. Here, we have investigated the effect of an inflammatory insult where neutrophils are recruited to the local aortic wall such as in aortic dissection (24). We have shown that the presence of neutrophil supernatants alters the formation of medin fibrils *in vitro.* Addition of neutrophil culture supernatants reduces the number of ThT positive fibrils formed, with fewer fibrils also observed by TEM. This lack of fibril formation is also associated with a reduction in cellular toxicity associated with the fibrils. Assessment of cellular viability was important to evaluate whether reduction of fibril formation could be considered a protective role or whether it increased the presence of pre-fibrillar or oligomeric intermediates with enhanced toxicity as observed previously for medin (25).

Following measurement of proteases present within our neutrophil supernatants we evaluated the effect of adding inhibitors for MMPs, serine and cysteine proteases. The presence of neutrophil degranulation supernatants in combination with inhibitors removed the cytotoxic protective effect offered by the neutrophils with greater cell toxicity observed for medin and neutrophil supernatants in the presence of all inhibitors compared with medin and neutrophil supernatants alone. GM6001 and AEBSF also showed a rescue of ThT fluorescence back to levels observed for medin alone, with fibrillar species observed by TEM. This suggests that both serine protease and MMP activity are likely contributing to inhibition of fibril formation by neutrophil supernatants. In contrast, presence of E64 showed an absence of fibril formation comparable with that observed when medin was incubated with neutrophil supernatants alone. However, cell viability data showed that the species produced have very different toxicity effects (~29% viability in the presence of neutrophil supernatants and E64, compared with ~87% for neutrophil supernatants alone). This suggests that while cysteine protease activity may not be responsible for the inhibition of fibril formation, the presence of neutrophil supernatants and E64 is generating highly toxic species. This may indicate that an E64 inhibitory target was preventing formation of these toxic species.

We have previously shown that medin can induce an inflammatory response in cells (15–18). Here we showed that the presence of neutrophil degranulation products can alter medin aggregation, and inhibition of MMPs and serine proteases can reverse the observed effect, suggesting that the action of these proteases is at least partially responsible for inhibition. One reason for this could be that MMPs and serine proteases present in neutrophil supernatants could break down/degrade medin, in turn preventing amyloid formation. Consistent with this, previous studies have shown that amyloid fibrils can trigger formation of NETs, in turn causing fibril fragmentation (26). Neutrophils have also been shown to enhance clearance of systemic amyloid deposits with reduced amyloid clearance observed in mice with depleted neutrophils (27). Here, we have treated the neutrophils for 1 h which isn’t long enough to stimulate NET production, even with potent inducers of NETs such as phorbol 12-myristate 13-acetate (PMA) which take up to 2 h (28). Instead, our treatment induces granule mobilisation to the plasma membrane then degranulation and release of proteases (29).

Medin caused increased production of MMP2 when added to aortic smooth muscle cells (4). It is proposed that MMP2 and other neutrophil-secreted proteases e.g. neutrophil elastase and cathepsins act by degrading the extracellular matrix within the aortic wall, in turn causing weakening and contributing to aneurysm and dissection. Data presented here implies cells may be attempting to protect against medin-insult by producing MMPs that act to prevent fibril formation. This further implicates the role of the inflammatory response in both protecting the body and promoting regeneration of homeostasis, while its dysregulation plays an important role in pathogenesis.

Inflammatory cells are found co-localised with amyloid deposits in many amyloid diseases (30, 31). The accumulation of amyloid fibrils causes local tissue damage with associated immune cell infiltration and production of proinflammatory cytokines (14). In turn, an inflammatory environment stimulates amyloid production, further contributing to dysfunction of the tissue/organ. Strategies targeting this inflammatory cycle and re-balancing it have been investigated as approaches to target amyloid accumulation in animal models of AD (32).

Non-steroidal anti-inflammatory drugs have been proposed as a therapeutic strategy in AD patients, albeit with inconsistent results (33). Indeed, suggesting that more specific targeting may be required to achieve a reduction in pathological effects related to amyloid formation. There are a range of neutrophil-targeting therapies that have been developed targeting many conditions including cancer, respiratory and autoimmune diseases (34, 35). In cancer, neutrophils have been shown to have both pro and anti-tumour functions with therapies targeting a range of neutrophil activities including neutrophil depletion, modulation of function, polarisation, recruitment and cytotoxicity. The varied therapies act to either activate or inhibit neutrophils depending on the specific neutrophil function or neutrophil-associated receptor they are designed to target. Neutrophil-activating therapies combining actions of TNF, CD40 agonist and tumour-binding antibodies have shown induction of neutrophil-mediated eradication of cancer in animal models and *in vitro* (36). Unsurprisingly given the variety of roles associated with neutrophils varying success has been observed from reaching FDA approval for psoriasis treatment, with clinical trials underway in cancer, while other targets failed to show any efficacy. The availability of these therapies with associated safety data provides avenues to explore neutrophil-specific targeting in diseases associated with AMA deposition.

There are limitations to this study that should be noted including the use of neutrophils from healthy donors, *in vitro* evaluation and small sample numbers. This study used samples from 2 donors based on the protease screen being able to evaluate n=4 samples only (n=2, T and UT). There is variability between the 2 donors as can be seen in the data presented in **Figure 2** but both donors had comparable effects on medin aggregation as observed in **Figure 1**, consistent with our findings in this proof-of-concept study. The neutrophils from healthy donors could contain different proteases with altered activity compared to neutrophils from diseased states, so may not fully replicate *in vivo* neutrophil activation from patients. The inhibitors used are broad-spectrum. For example, E64 has is a robust inhibitor of most cysteine proteinases, including cathepsin K, L S, B, H papain and calpain. More specific inhibitors and combinations would be required to elucidate exact mechanisms of action and propose potential avenues for increasing or decreasing specific activity. However, given the relative lack of knowledge regarding the role of inflammation and fibril formation in conditions where medin could be associated this initial study does provide an avenue for further exploration as to the role of neutrophil-associated inflammation within medin fibril formation.

We have investigated fibrils formed in the presence of neutrophil supernatants and how the presence of the supernatants alters the properties of the aggregates formed (morphology, kinetics and toxicity). The physiological condition where this would occur is when the inflammatory response occurs prior to and/or during medin aggregation. The initiating factor(s) for cleavage of medin from MFGE8 or subsequent aggregation are currently unclear. However, inflammation associated processes including the production of proteases and cytokines are highly likely to be involved. Therefore, we believe that presence of neutrophil-associated factors formed upon degranulation as we have in our experimental set-up here during aggregation represents a potential physiologically relevant situation.

We have previously shown that addition of medin to HUVECs can increase superoxide and decrease bioavailable nitric oxide production providing a reactive oxygen species (ROS) rich environment which can produce peroxynitrite, in turn modifying tyrosines on medin (22). Nitrated medin showed a reduction in ThT fluorescence *in vitro*, however fibrils were still formed but with altered morphology with increased cell toxicity. This proposed an altered ROS environment as a possibility for enhancing medin-associated cell toxicity. Neutrophils can produce ROS, with response dependent upon their activation state and maturity (23). We measured ROS production following fMLP-stimulation for neutrophils alone (untreated), or in the presence of medin or the cytokine tumour necrosis factor α (TNFα) for 1 h (**Supplementary Figure S3**). Addition of medin to neutrophils suggested that medin acted like a cytokine to “prime” neutrophils for ROS production in the same way TNFα does. In contrast, pre-formed medin fibrils (in the absence of neutrophil supernatants) did not show ROS production. This provides an additional avenue for exploration in AMA-associated pathogenesis of the interplay between neutrophils and ROS and effect on fibril formation.

We show here that there is an interplay between neutrophils produced as part of the body’s natural immune response and medin aggregation and that this interaction can be modulated by proteolytic inhibition. Interestingly, neutrophils appear to be protective against medin fibril formation *in vitro*, suggesting a beneficial role protecting against pathogenicity associated with fibrils. We also show that stimulated neutrophils produce factors that are inherently toxic to cells, suggesting that there may be an interplay between the apparent protective effect observed here and inherent toxicity in a stimulated environment. We know that inflammation becomes dysregulated in diseases such as those associated with medin aggregation suggesting that controlled targeting of the neutrophil response could be a valid therapeutic approach in these conditions.

## Data Availability

The original contributions presented in the study are included in the article/**supplementary material**. Further inquiries can be directed to the corresponding author.

## Supplementary material

Supplementary Figure 1 Representative image of cytospin showing neutrophils (purple polymorphic nucleus with clear cytoplasm) with minor contamination (<3%) from eosinophils (purple nucleus with orange/pink cytoplasm). Image shown at x25 magnification.

Supplementary Figure 2 Thioflavin T fluorescence of pre-formed medin fibrils in the presence of neutrophil supernatants. Thioflavin T fluorescence of 20 µM pre-formed medin fibrils alone (grey) in PBS, pH7.4 and in the presence of **(A)** treated and **(B)** untreated neutrophil supernatants at 20% v/v over 99 h. Data is presented as mean traces for n=3 per condition. **(C)** TEM images of endpoint ThT samples. Scale bar in 500 nm as shown.

Supplementary Figure 3 Medin primed neutrophils produce reactive oxygen species (ROS) following stimulation. Neutrophils (5x10^6^) were incubated for 1 h alone (untreated, UT), with medin (300 µM) or TNFα (10 ng/mL). ROS production was stimulated by fMLP (receptor-dependent) and measured by luminol-enhanced chemiluminescence (mean trace shown from n=2 experiments).

## References

[1] Peng S Glennert J Westermark P . Medin-amyloid: a recently characterized age-associated arterial amyloid form affects mainly arteries in the upper part of the body. Amyloid. (2005) 12:96–102. doi: 10.1080/13506120500107006, PMID: 16011985

[2] Mucchiano G Cornwell GG 3rd Westermark P . Senile aortic amyloid. Evidence for two distinct forms of localized deposits. Am J Pathol. (1992) 140:871–7., PMID: 1562050 PMC1886375

[3] Davies HA Caamaño-Gutiérrez E Chim YH Field M Nawaytou O Ressel L . Idiopathic degenerative thoracic aneurysms are associated with increased aortic medial amyloid. Amyloid. (2019) 26:148–55. doi: 10.1080/13506129.2019.1625323, PMID: 31210552 PMC6816484

[4] Peng S Larsson A Wassberg E Gerwins P Thelin S Fu X . Role of aggregated medin in the pathogenesis of thoracic aortic aneurysm and dissection. Lab Invest. (2007) 87:1195–205. doi: 10.1038/labinvest.3700679, PMID: 17906662

[5] Wagner J Degenhardt K Veit M Louros N Konstantoulea K Skodras A . Medin co-aggregates with vascular amyloid-β in Alzheimer’s disease. Nature. (2022) 612:123–31. doi: 10.1038/s41586-022-05440-3, PMID: 36385530 PMC9712113

[6] Migrino RQ Karamanova N Truran S Serrano GE Davies HA Madine J . Cerebrovascular medin is associated with Alzheimer’s disease and vascular dementia. Alzheimers Dement (Amst). (2020) 12:e12072. doi: 10.1002/dad2.12072, PMID: 32875054 PMC7447901

[7] Häggqvist B Näslund J Sletten K Westermark GT Mucchiano G Tjernberg LO . Medin: An integral fragment of aortic smooth muscle cell-produced lactadherin forms the most common human amyloid. Proc Natl Acad Sci. (1999) 96:8669–74. doi: 10.1073/pnas.96.15.8669, PMID: 10411933 PMC17574

[8] Kolaczkowska E Kubes P . Neutrophil recruitment and function in health and inflammation. Nat Rev Immunol. (2013) 13:159–75. doi: 10.1038/nri3399, PMID: 23435331

[9] Michalska M Grochowiecki T Jakimowicz T Nazarewski S . A review of the impact of neutrophils and neutrophil extracellular traps (NETs) on the development of aortic aneurysms in animal and human studies. Med Sci Monit. (2021) 27:e935134. doi: 10.12659/MSM.935134, PMID: 34961758 PMC8720181

[10] Kurihara T Shimizu-Hirota R Shimoda M Adachi T Shimizu H Weiss SJ . Neutrophil-derived matrix metalloproteinase 9 triggers acute aortic dissection. Circulation. (2012) 126:3070–80. doi: 10.1161/CIRCULATIONAHA.112.097097, PMID: 23136157

[11] Lareyre F Raffort J Le D Chan HL Le Houerou T Cochennec F . High neutrophil to lymphocyte ratio is associated with symptomatic and ruptured thoracic aortic aneurysm. Angiology. (2018) 69:686–91. doi: 10.1177/0003319717751758, PMID: 29334754

[12] Kalkan ME Kalkan AK Gündes A Yanartas M Oztürk S Gurbuz AS . Neutrophil to lymphocyte ratio: a novel marker for predicting hospital mortality of patients with acute type A aortic dissection. Perfusion. (2017) 32:321–7. doi: 10.1177/0267659115590625, PMID: 26467992

[13] Mohammadi A Mohammadi M Almasi-Dooghaee M Mirmosayyeb O . Neutrophil to lymphocyte ratio in Alzheimer’s disease: A systematic review and meta-analysis. PloS One. (2024) 19:e0305322. doi: 10.1371/journal.pone.0305322, PMID: 38917167 PMC11198755

[14] Foguel D Azevedo E . The role of inflammation in amyloid diseases. In: Kurouski D , editor. Amyloid Diseases. IntechOpen, Rijeka (2018).

[15] Karamanova N Morrow KT Maerivoet A Madine J Li M Migrino RQ . Medin induces pro-inflammatory activation of human brain vascular smooth muscle cells. Physiol Rep. (2025) 13:e70418. doi: 10.14814/phy2.70418, PMID: 40501029 PMC12159308

[16] Migrino RQ Davies HA Truran S Karamanova N Franco DA Beach TC . Amyloidogenic medin induces endothelial dysfunction and vascular inflammation through the receptor for advanced glycation endproducts. Cardiovasc Res. (2017) 113:1389–402. doi: 10.1093/cvr/cvx135, PMID: 28859297 PMC6676393

[17] Karamanova N Truran S Serrano GE Beach TG Madine J Weissig W . Endothelial immune activation by medin: potential role in cerebrovascular disease and reversal by monosialoganglioside-containing nanoliposomes. J Am Heart Assoc. (2020) 9:e014810. doi: 10.1161/JAHA.119.014810, PMID: 31928157 PMC7033828

[18] Zhang Y Karamanova N Morrow KT Madine J Truran S Lozoya M . Transcriptomic analyses reveal proinflammatory activation of human brain microvascular endothelial cells by aging-associated peptide medin and reversal by nanoliposomes. Sci Rep. (2023) 13:18802. doi: 10.1038/s41598-023-45959-7, PMID: 37914766 PMC10620412

[19] Shirley S Maerivoet A Wright HL Field M Madine J . Systematic review and meta-analysis of admission inflammatory biomarkers for evaluating prognosis in acute type A aortic dissection. Aorta (Stamford). (2025) 13:079–93. doi: 10.1055/a-2693-4070, PMID: 41056983 PMC13300749

[20] Mitchell TS Moots RJ Wright HL . Janus kinase inhibitors prevent migration of rheumatoid arthritis neutrophils towards interleukin-8, but do not inhibit priming of the respiratory burst or reactive oxygen sp*ecies production*. Clin Exp Immunol. (2017) 189:250–8. doi: 10.1111/cei.12970, PMID: 28369741 PMC5508336

[21] Martin-Martin B Tovell V Dahlmann-Noor AH Khaw PT Bailly M . The effect of MMP inhibitor GM6001 on early fibroblast-mediated collagen matrix contraction is correlated to a decrease in cell protrusive activity. Eur J Cell Biol. (2011) 90:26–36. doi: 10.1016/j.ejcb.2010.09.008, PMID: 21040999 PMC7611814

[22] Davies HA Phelan MM Wilkinson MC Migrino RQ Truran S Franco DA . Oxidative stress alters the morphology and toxicity of aortic medial amyloid. Biophys J. (2015) 109:2363–70. doi: 10.1016/j.bpj.2015.10.034, PMID: 26636947 PMC4675884

[23] Montaldo E Lusito E Bianchessi V Caronni N Scala S Basso-Ricci L . Cellular and transcriptional dynamics of human neutrophils at steady state and upon stress. Nat Immunol. (2022) 23:1470–83. doi: 10.1038/s41590-022-01311-1, PMID: 36138183 PMC7615267

[24] Wu D Choi JC Sameri A Minard CG Coselli JS Shen YH . Inflammatory cell infiltrates in acute and chronic thoracic aortic dissection. Aorta (Stamford). (2013) 1:259–67. doi: 10.12945/j.aorta.2013.13-044, PMID: 26798703 PMC4682718

[25] Younger S Jang H Davies HA Niemiec MJ Garcia JGN Nussinov R . Medin oligomer membrane pore formation: A potential mechanism of vascular dysfunction. Biophys J. (2020) 118:2769–82. doi: 10.1016/j.bpj.2020.04.026, PMID: 32402244 PMC7264854

[26] Azevedo EP Guimarães-Costa AB Torezani GS Braga CA Palhano FL Kelly JW . Amyloid fibrils trigger the release of neutrophil extracellular traps (NETs), causing fibril fragmentation by NET-associated elastase. J Biol Chem. (2012) 287:37206–18. doi: 10.1074/jbc.M112.369942, PMID: 22918834 PMC3481320

[27] Hancock TJ Vlasyuk M Foster JS Macy S Wooliver DC Balachandran M . Neutrophils enhance the clearance of systemic amyloid deposits in a murine amyloidoma model. Front Immunol. (2024) 15:1487250. doi: 10.3389/fimmu.2024.1487250, PMID: 39600710 PMC11588727

[28] Chapman EA Lyon M Simpson D Mason D Beynon RJ Moots RJ . Caught in a trap? Proteomic analysis of neutrophil extracellular traps in rheumatoid arthritis and systemic lupus erythematosus. Front Immunol. (2019) 10. doi: 10.3389/fimmu.2019.00423, PMID: 30915077 PMC6421309

[29] Cross AL Hawkes J Wright HL Moots RJ Edwards SW . APPA (apocynin and paeonol) modulates pathological aspects of human neutrophil function, without supressing antimicrobial ability, and inhibits TNFα expression and signalling. Inflammopharmacology. (2020) 28:1223–35. doi: 10.1007/s10787-020-00715-5, PMID: 32383062 PMC7525285

[30] Farfara D Lifshitz V Frenkel D . Neuroprotective and neurotoxic properties of glial cells in the pathogenesis of Alzheimer’s disease. J Cell Mol Med. (2008) 12:762–80. doi: 10.1111/j.1582-4934.2008.00314.x, PMID: 18363841 PMC4401126

[31] McGeer PL Itagaki S Boyes BE McGeer EG . Reactive microglia are positive for HLA-DR in the substantia nigra of Parkinson’s and Alzheimer’s disease brains. Neurology. (1988) 38:1285–91. doi: 10.1212/WNL.38.8.1285, PMID: 3399080

[32] Guillot-Sestier MV Doty KR Town T . Innate immunity fights alzheimer’s disease. Trends Neurosci. (2015) 38:674–81. doi: 10.1016/j.tins.2015.08.008, PMID: 26549882 PMC4641041

[33] Szekely CA Zandi PP . Non-steroidal anti-inflammatory drugs and Alzheimer’s disease: the epidemiological evidence. CNS Neurol Disord Drug Targets. (2010) 9:132–9. doi: 10.2174/187152710791012026, PMID: 20205647

[34] Kwak JW Houghton AM . Targeting neutrophils for cancer therapy. Nat Rev Drug Discov. (2025) 24:666–84. doi: 10.1038/s41573-025-01210-8, PMID: 40374764 PMC13232631

[35] Stockley R De Soyza A Gunawardena K Perrett J Forsman-Semb K Entwistle N . Phase II study of a neutrophil elastase inhibitor (AZD9668) in patients with bronchiectasis. Respir Med. (2013) 107:524–33. doi: 10.1016/j.rmed.2012.12.009, PMID: 23433769

[36] Linde IL Prestwood TR Qiu J Pilarowski G Linde MH Zhang X . Neutrophil-activating therapy for the treatment of cancer. Cancer Cell. (2023) 41:356–372.e10. doi: 10.1016/j.ccell.2023.01.002, PMID: 36706760 PMC9968410

