# Supplementary material for: Neutrophils and aortic medial amyloid: mutually beneficial or a dangerous combination?
Source: Front Immunol. 2026 Feb 6;17:1699039. doi: 10.3389/fimmu.2026.1699039 (PMC12920188; doi:10.3389/fimmu.2026.1699039)
Supplement: Supplementary Figure 1 — Representative image of cytospin showing neutrophils (purple polymorphic nucleus with clear cytoplasm) with minor contamination (<3%) from eosinophils (purple nucleus with orange/pink cytoplasm). Image shown at x25 magnification. [file DataSheet1.docx]

**Supplementary Information**

**
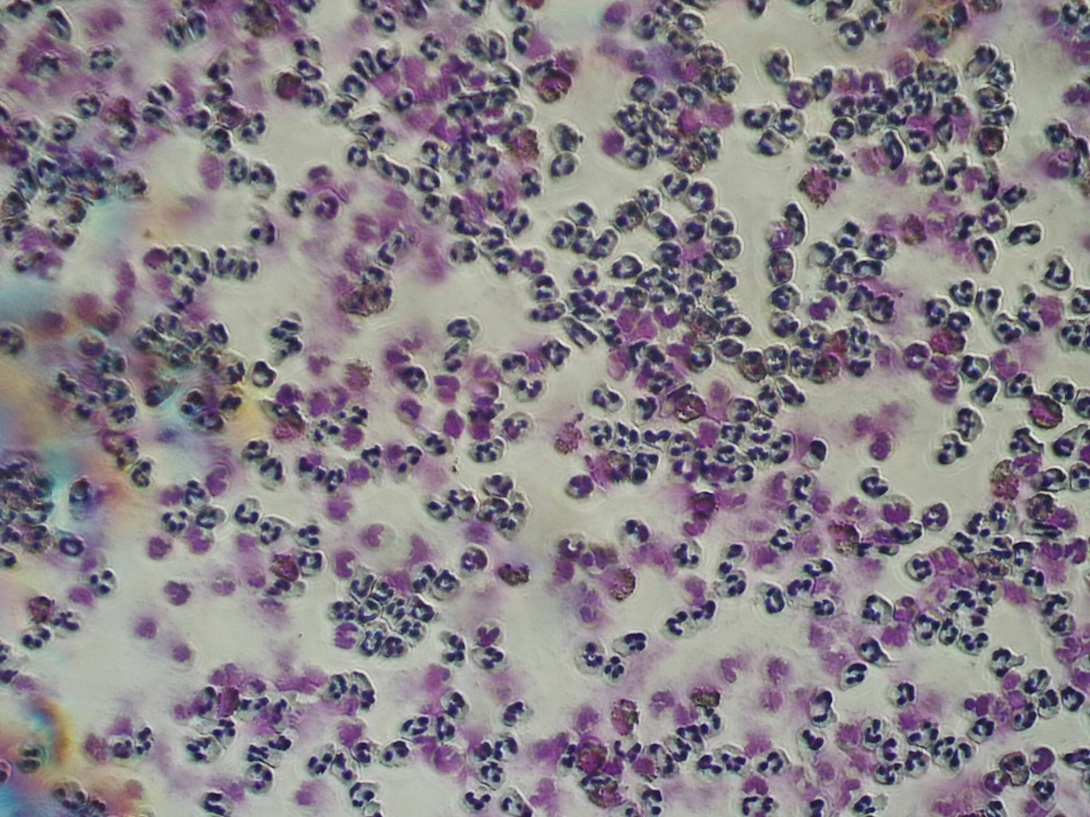
**

**Figure S1: Representative image of cytospin showing neutrophils (purple polymorphic nucleus with clear cytoplasm) with minor contamination (<3%) from eosinophils (purple nucleus with orange/pink cytoplasm).** Image shown at x25 magnification.

**
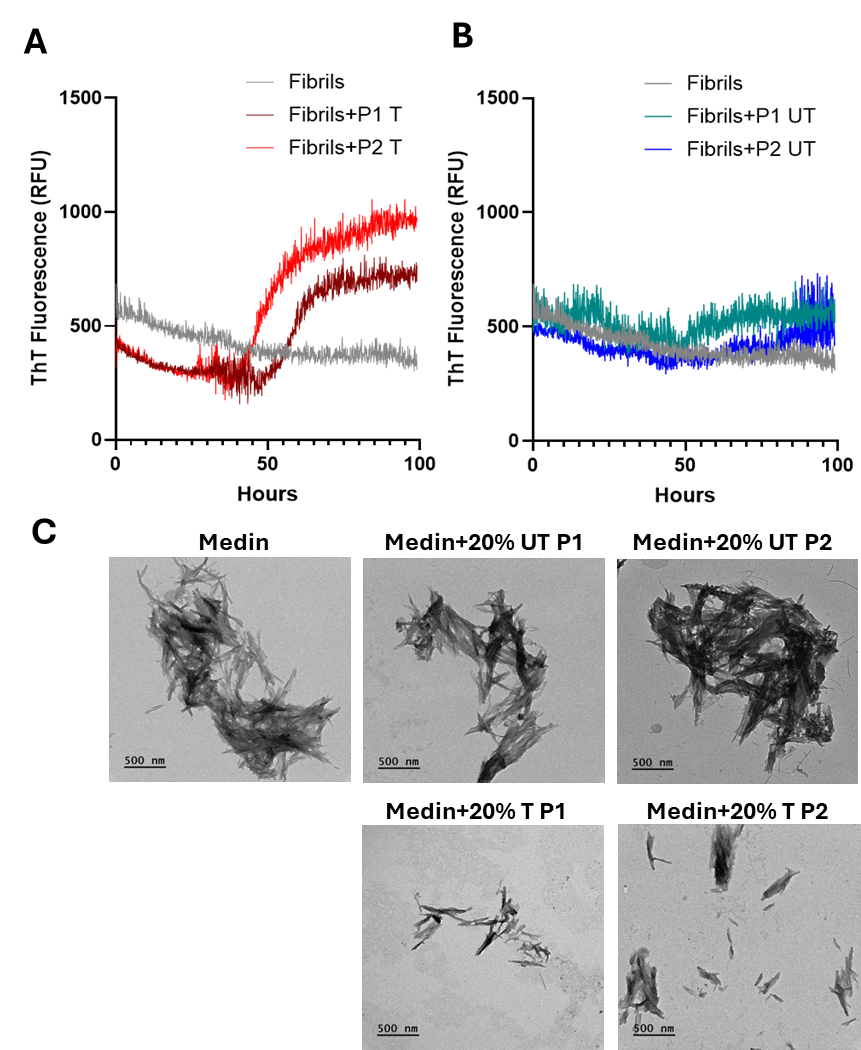
**

**Figure S2: Thioflavin T fluorescence of pre-formed medin fibrils in the presence of neutrophil supernatants.** Thioflavin T fluorescence of 20 µM pre-formed medin fibrils alone (grey) in PBS, pH7.4 and in the presence of A) treated and B) untreated neutrophil supernatants at 20% v/v over 99 h. Data is presented as mean traces for n=3 per condition. C) TEM images of endpoint ThT samples. Scale bar in 500 nm as shown.


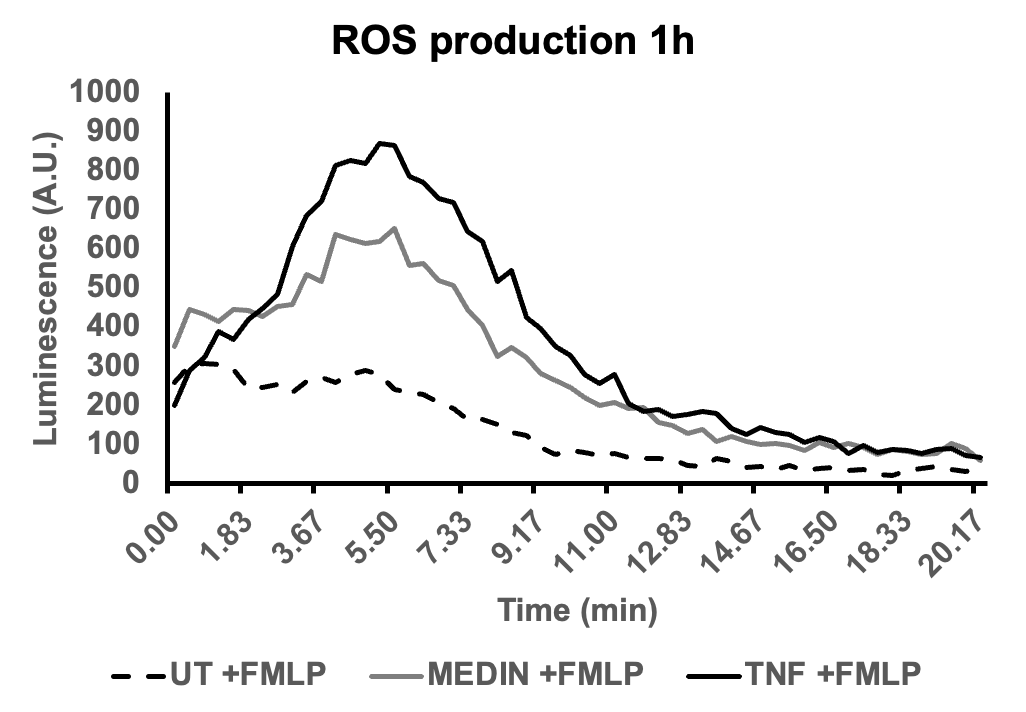


**Figure S3: Medin primed neutrophils produce reactive oxygen species (ROS) following stimulation.** Neutrophils (5x10^6^) were incubated for 1 h alone (untreated, UT), with medin (300 µM) or TNFα (10 ng/mL). ROS production was stimulated by fMLP (receptor-dependent) and measured by luminol-enhanced chemiluminescence (mean trace shown from n=2 experiments).

**Supplementary Methods**

*Cytospin Preparation and Imaging*

Cytospins were prepared by diluting 100,000 cells in 200 µL PBS (EDTA), loading into a cytospin chamber and centrifuging for 5 min at 5000 rpm in a Shandon Cytospin cytocentrifuge.  Slides were stained with Rapid Romanowsky stain and imaged on a light microscope at x25 magnification.

*Thioflavin T pre-formed fibrils*

Fibrils were formed by incubating 50 µM medin in PBS at 37°C for 7 days with shaking. Fibrils were diluted to 20 µM in PBS in a 96-well black-walled, clear-bottomed microplate and incubated in a Flexstation 3 microplate reader alone or in the presence of 20 % v/v of untreated or treated neutrophils and 2 µM ThT at 37°C under quiescent conditions with 5 s shaking every 5 mins for up to 99 hours. Experiments were carried out using neutrophils isolated from 2 donors with 3 wells per condition. Data were recorded every 5 min using bottom read mode, with excitation at 440 nm and emission at 490 nm.

*Neutrophil Reactive Oxygen Species (ROS) production*

Isolated neutrophils (5x10^6^/mL) were incubated in screw-top eppendorfs at 37^o^C with continuous rotation for 1 h. Neutrophils were stimulated with medin monomer (300μM), TNFα(10 ng/mL) or left untreated. ROS production was measured by diluting 2x10^5^ neutrophils in 200 μL HBSS (total volume) containing 10 μM luminol in a white, low-adhesion 96-well plate (Corning). ROS production was stimulated with 1 μM fMLP and luminol-enhanced chemiluminescence measured continuously for up to 30 min at 37^o^C in a Tecan plate reader.
